# 9-Furfuryl­idene-2,3-dimethyl-6,7,8,9-tetrahydro-4*H*-­thieno[2′,3′:4,5]pyrimidino[1,2-*a*]pyridin-4-one

**DOI:** 10.1107/S1600536810004101

**Published:** 2010-02-06

**Authors:** Khurshed A. Bozorov, Burkhon Zh. Elmuradov, Rasul Ya. Okmanov, Bakhodir Tashkhodjaev, Khusnutdin M. Shakhidoyatov

**Affiliations:** a S. Yunusov Institute of the Chemistry of Plant Substances, Academy of Sciences of Uzbekistan, Mirzo Ulugbek Str. 77, Tashkent 100170, Uzbekistan

## Abstract

The title compound, C_17_H_16_N_2_O_2_S, was obtained by condensation of 2,3-dimethyl­thieno[2′,3′:4,5]pyrimidino[1,2-*a*]pyridin-4-one with furfural in the presence of sodium hydroxide. One of the methyl­ene groups of the tetra­hydro­pyrido ring is disordered over two positions in a 0.87 (1):0.13 (1) ratio. The thieno[2,3-*d*]pyrimidin-4-one unit and the furan ring are both planar (r.m.s. deviation = 0.535 Å), and coplanar with each other, forming a dihedral angle of 5.4 (1)°. Four weak inter­molecular hydrogen bonds (C—H⋯O and C—H⋯N) are observed in the structure, which join mol­ecules into a network parallel to (101).

## Related literature

For the synthesis of thieno[2,3-*d*]pyrimidin-4-ones and their derivatives, see: Melik-Ogandzhanyan *et al.* (1985 ▶); Csukonyi *et al.* (1986 ▶); Shvedov *et al.* (1975 ▶); Shakhidoyatov (1983 ▶); Gevald *et al.* (1966 ▶); Kapustina *et al.* (1992 ▶); Peet *et al.* (1986 ▶); Shodiyev *et al.* (1993 ▶); Bozorov *et al.* (2009 ▶). For the physiological activity of thieno[2,3-*d*]pyrimidin-4-ones and their derivatives, see: Kapustina *et al.* (1992 ▶); Blaskiewich *et al.* (1975 ▶); Wähäla *et al.* (2005 ▶); Lilienkampf *et al.* (2007 ▶); Han *et al.* (2007 ▶); Moore *et al.* (2006 ▶). For weak hydrogen bonds in alkaloids, see: Rajnikant *et al.* (2005 ▶). For bond-length data, see: Allen *et al.* (1987 ▶). 
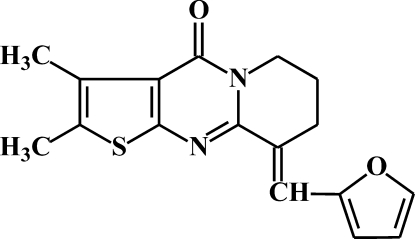


## Experimental

### 

#### Crystal data

C_17_H_16_N_2_O_2_S
*M* *_r_* = 312.39
Monoclinic, 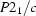

*a* = 16.569 (3) Å
*b* = 11.034 (2) Å
*c* = 8.2775 (17) Å
β = 93.12 (3)°
*V* = 1511.1 (5) Å^3^
*Z* = 4
Cu *K*α radiation
μ = 1.98 mm^−1^
*T* = 295 K
0.70 × 0.25 × 0.25 mm

#### Data collection

Stoe Stadi-4 four-circle diffractometer
Absorption correction: ψ scan (North *et al.*, 1968 ▶) *T*_min_ = 0.749, *T*_max_ = 0.994
2398 measured reflections
2252 independent reflections
1875 reflections with *I* > 2σ(*I*)
θ_max_ = 60.0°
3 standard reflections every 60 min  intensity decay: 8.8%

#### Refinement

*R*[*F* ^2^ > 2σ(*F* ^2^)] = 0.046
*wR*(*F* ^2^) = 0.126
*S* = 1.06
2252 reflections
212 parameters
H-atom parameters constrained
Δρ_max_ = 0.21 e Å^−3^
Δρ_min_ = −0.20 e Å^−3^

### 

Data collection: *STADI4* (Stoe & Cie, 1997 ▶); cell refinement: *STADI4*; data reduction: *X-RED* (Stoe & Cie, 1997 ▶); program(s) used to solve structure: *SHELXS97* (Sheldrick, 2008 ▶); program(s) used to refine structure: *SHELXL97* (Sheldrick, 2008 ▶); molecular graphics: *XP* in *SHELXTL* (Sheldrick, 2008 ▶); software used to prepare material for publication: *SHELXL97*.

## Supplementary Material

Crystal structure: contains datablocks I, global. DOI: 10.1107/S1600536810004101/zl2270sup1.cif

Structure factors: contains datablocks I. DOI: 10.1107/S1600536810004101/zl2270Isup2.hkl

Additional supplementary materials:  crystallographic information; 3D view; checkCIF report

## Figures and Tables

**Table 1:** Hydrogen-bond geometry (Å, °)

*D*—H⋯*A*	*D*—H	H⋯*A*	*D*⋯*A*	*D*—H⋯*A*
C3′—H3′*A*⋯O1′^i^	0.93	2.58	3.442 (3)	154
C4′—H4′*A*⋯N1^i^	0.93	2.66	3.568 (3)	166
C5*A*—H5*AA*⋯O1^ii^	0.96	2.55	3.486 (4)	166
C6*A*—H6*AA*⋯O1^ii^	0.96	2.62	3.571 (4)	171

Symmetry codes: (i) 
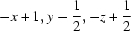
; (ii) 
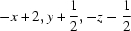
.

**Fig. 1.**
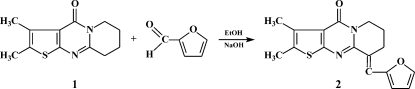
Reaction scheme.

**Fig. 2.**
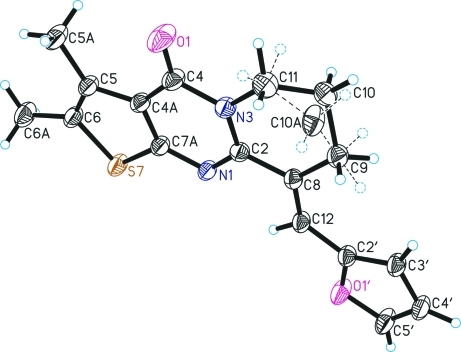
An Ortep sty;e plot of the structure and the numbering of atoms in (I) (dashed atoms indicate equivalent positions of disordeed atoms).

**Fig. 3.**
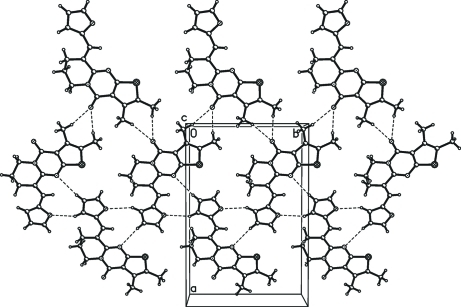
Packing view of the title compound showing the H-bonding networks in the crystal. Minor moiety disordered atoms are omitted for clarity.

## supplementary crystallographic information

### Comment

Among heterocyclic compounds the thieno[2,3-*d*]pyrimidin-4-ones (Melik-Ogandzhanyan *et al.*, 1985; Csukonyi *et al.*, 1986; Shvedov *et al.*, 1975; Shakhidoyatov 1983; Gevald *et al.*, 1966; Kapustina *et al., *1992; Peet *et al., *1986; Shodiyev *et al., *1993) make up a large group of substances that have various physiological activities (Kapustina *et al., *1992; Blaskiewich *et al., *1975; Wähäla *et al., *2005; Lilienkampf *et al., *2007; Han *et al.*, 2007; Moore *et al.*, 2006).

Condensation of 2,3-dimethylthieno[2',3':4,5]pyrimidino[1,2-*a*]pyridin-4-one with aromatic and heterocyclic aldehydes leads to the formation of new 8-aryliden derivatives. With this purpose in mind the reaction of 2,3-dimethylthieno[2',3':4,5]pyrimidino[1,2-*a*]pyridin-4-one with furfural was carried by boiling of equimolar amounts of the initial reagents over 4 hours in ethanol in the presence of sodium hydroxide (Bozorov *et al.*, 2009) (Figure 1).

The structure of the synthesized compound has been investigated by ^1^H NMR and XRD analysis. Figure 2 shows an ortep style plot of the molecular structure of the title compound. One of the methylene groups of the tetrahydropyrido ring (C10, C10A) is disordered over two positions. Refinement of the structure yielded an occupancy ratio of the disordered atoms (*i.e.* two conformers) of 0.87 (1):0.13 (1).

The π-electronic system of the thiophene, furan and pyrimidinone rings participate in conjugation with the π electrons of the nitrogen atoms as can be seen from the appreciable change of the bond lengths of valence bonds C4═O1 (1.226 (3) Å), C2—C8 (1.479 (3) Å), C12—C2' (1.437 (3) Å) from their standard values (Allen *et al.*, 1987) and from the coplanarity of the thieno[2,3-*d*]pyrimidin-4-one moiety with the furan ring.

In the crystal structure of the title compound weak intermolecular C—H···X hydrogen bonds (Table 1) are observed as it is often the case in alkaloids (Rajnikant *et al.*, 2005). Of them C3'—H···O1' and C4'—H···N1 lead to the formation of infinite chains, C5A—H···O1 and C6A—H···O1 join these chains in a flat network (Figure 3. and Table 1) parallel to the (1 0 1) plane.

### Experimental

0.02 g sodium hydroxide (0.5 mmole) was dissolved in 5 ml ethanol, and 0.234 g (1 mmole) of 2,3-dimethylthieno[2',3':4,5]pyrimidino[1,2-*a*]pyridin-4-one and 0.106 g (0.092 ml, d═1.1598 g/ml, 1.1 mmole) furfural were added (Figure 1). The mixture was heated to reflux on a water bath for 4 hours. The solvent was distilled off and the residue was recrystallized from a mixture of solvents – benzene: cyclohexane – 5:1. 0.26 g (83.4 %) of the title compound were obtained in the reaction. m.p. 449–451 K.

Yellow crystals suitable for X-ray analysis were obtained from a mixture of benzene and hexane (2:1) at room temperature.

^1^H NMR (400 MHz, CDCl_3_): 7.83 (1*H*, t, J═2.22 Hz, H-12), 7.48 (1*H*, d, J═1.98 Hz, H-3'), 6.56 (1*H*, d, J═3.46 Hz, H-5'), 6.45 (1*H*, dd, J═1.98 Hz, J═3.46 Hz, H-4'), 4.07 (2*H*, t, J═5.93 Hz, CH_2_-11), 2.97 (2*H*, td, J═2.22 Hz, J═6.68 Hz, CH_2_-9), 2.42 (3*H*, d, J═0.75 Hz, CH_3_-5), 2.32 (3*H*, d, J═0.75 Hz, CH_3_-6), 1.94-1.99 (2*H*, m, CH_2_-10).

### Refinement

The H atoms bonded to C atoms were placed geometrically (with C—H distances of 0.98 Å for CH; 0.97 Å for CH_2_; 0.96 Å for CH_3_; and 0.93 Å for C_ar_) and included in the refinement in a riding motion approximation with U_iso_=1.2U_eq_(C) [U_iso_=1.5U_eq_(C) for methyl H atoms].

### Crystal data

**Table:**

C_17_H_16_N_2_O_2_S	*F*(000) = 656
*M**_r_* = 312.39	*D*_x_ = 1.373 Mg m^−^^3^
Monoclinic, *P*2_1_/*c*	Melting point: 448(3) K
Hall symbol: -P 2ybc	Cu *K*α radiation, λ = 1.54184 Å
*a* = 16.569 (3) Å	Cell parameters from 14 reflections
*b* = 11.034 (2) Å	θ = 10–20°
*c* = 8.2775 (17) Å	µ = 1.98 mm^−^^1^
β = 93.12 (3)°	*T* = 295 K
*V* = 1511.1 (5) Å^3^	Prizmatic, yellow
*Z* = 4	0.70 × 0.25 × 0.25 mm

### Data collection

**Table:**

Stoe Stadi-4 four-circle diffractometer	1875 reflections with *I* > 2σ(*I*)
Radiation source: fine-focus sealed tube	*R*_int_ = 0.000
graphite	θ_max_ = 60.0°, θ_min_ = 2.7°
Scan width (ω) = 1.56 – 1.80, scan ratio 2θ:ω = 1.00 I(Net) and sigma(I) calculated according to Blessing (1987)	*h* = 0→18
Absorption correction: ψ scan (North *et al.*, 1968)	*k* = −12→0
*T*_min_ = 0.749, *T*_max_ = 0.994	*l* = −9→9
2398 measured reflections	3 standard reflections every 60 min
2252 independent reflections	intensity decay: 8.8%

### Refinement

**Table:**

Refinement on *F*^2^	Secondary atom site location: difference Fourier map
Least-squares matrix: full	Hydrogen site location: inferred from neighbouring sites
*R*[*F*^2^ > 2σ(*F*^2^)] = 0.046	H-atom parameters constrained
*wR*(*F*^2^) = 0.126	*w* = 1/[σ^2^(*F*_o_^2^) + (0.0607*P*)^2^ + 0.8817*P*] where *P* = (*F*_o_^2^ + 2*F*_c_^2^)/3
*S* = 1.06	(Δ/σ)_max_ = 0.004
2252 reflections	Δρ_max_ = 0.21 e Å^−^^3^
212 parameters	Δρ_min_ = −0.20 e Å^−^^3^
0 restraints	Extinction correction: *SHELXL97* (Sheldrick, 2008), Fc^*^=kFc[1+0.001xFc^2^λ^3^/sin(2θ)]^-1/4^
Primary atom site location: structure-invariant direct methods	Extinction coefficient: 0.0065 (6)

### Special details

**Table:**

Experimental. ψ Scan Reflections used µ * R = 0.00 H K L, θ, χ, I_min_/I_max_: 2 0 0, 21.5, 84.7, 0.699
Geometry. All esds (except the esd in the dihedral angle between two l.s. planes) are estimated using the full covariance matrix. The cell esds are taken into account individually in the estimation of esds in distances, angles and torsion angles; correlations between esds in cell parameters are only used when they are defined by crystal symmetry. An approximate (isotropic) treatment of cell esds is used for estimating esds involving l.s. planes.
Refinement. Refinement of *F*^2^ against ALL reflections. The weighted *R*-factor wR and goodness of fit *S* are based on *F*^2^, conventional *R*-factors *R* are based on *F*, with *F* set to zero for negative *F*^2^. The threshold expression of *F*^2^ > σ(*F*^2^) is used only for calculating *R*-factors(gt) etc. and is not relevant to the choice of reflections for refinement. *R*-factors based on *F*^2^ are statistically about twice as large as those based on *F*, and *R*- factors based on ALL data will be even larger.

### Fractional atomic coordinates and isotropic or equivalent isotropic displacement parameters (Å^2^)

**Table:**

	*x*	*y*	*z*	*U*_iso_*/*U*_eq_	Occ. (<1)
O1	0.90729 (13)	0.21420 (19)	−0.1624 (3)	0.0835 (8)	
S7	0.77689 (4)	0.60019 (6)	−0.01745 (10)	0.0595 (3)	
N1	0.71466 (12)	0.37806 (19)	0.0356 (3)	0.0467 (6)	
C2	0.72119 (14)	0.2591 (2)	0.0249 (3)	0.0418 (6)	
N3	0.78599 (12)	0.20424 (19)	−0.0432 (3)	0.0458 (5)	
C4	0.85116 (15)	0.2697 (3)	−0.1063 (3)	0.0518 (7)	
C4A	0.84293 (15)	0.3988 (2)	−0.0951 (3)	0.0449 (6)	
C5	0.89660 (15)	0.4932 (2)	−0.1467 (3)	0.0487 (6)	
C5A	0.97383 (17)	0.4694 (3)	−0.2303 (4)	0.0679 (9)	
H5AA	0.9986	0.5451	−0.2562	0.102*	
H5AB	0.9618	0.4245	−0.3280	0.102*	
H5AC	1.0102	0.4235	−0.1599	0.102*	
C6	0.86857 (16)	0.6048 (3)	−0.1123 (3)	0.0531 (7)	
C6A	0.90635 (19)	0.7268 (3)	−0.1409 (4)	0.0709 (9)	
H6AA	0.9540	0.7164	−0.2005	0.106*	
H6AB	0.9207	0.7645	−0.0388	0.106*	
H6AC	0.8684	0.7772	−0.2016	0.106*	
C7A	0.77579 (14)	0.4433 (2)	−0.0239 (3)	0.0450 (6)	
C8	0.65649 (15)	0.1844 (2)	0.0904 (3)	0.0421 (6)	
C9	0.65812 (17)	0.0487 (2)	0.0699 (3)	0.0538 (7)	
H9A	0.6036	0.0194	0.0455	0.065*	0.867 (11)
H9B	0.6782	0.0114	0.1703	0.065*	0.867 (11)
H9C	0.6373	0.0109	0.1648	0.065*	0.133 (11)
H9D	0.6229	0.0265	−0.0228	0.065*	0.133 (11)
C10	0.7120 (3)	0.0122 (3)	−0.0664 (6)	0.0553 (13)	0.867 (11)
H10A	0.7188	−0.0751	−0.0657	0.066*	0.867 (11)
H10B	0.6857	0.0348	−0.1696	0.066*	0.867 (11)
C10A	0.7417 (14)	0.0010 (18)	0.046 (5)	0.059 (9)	0.133 (11)
H10C	0.7690	−0.0087	0.1520	0.071*	0.133 (11)
H10D	0.7364	−0.0790	−0.0017	0.071*	0.133 (11)
C11	0.79243 (19)	0.0704 (3)	−0.0502 (4)	0.0619 (8)	
H11A	0.8235	0.0477	−0.1414	0.074*	0.867 (11)
H11B	0.8211	0.0414	0.0476	0.074*	0.867 (11)
H11C	0.7820	0.0459	−0.1619	0.074*	0.133 (11)
H11D	0.8479	0.0487	−0.0200	0.074*	0.133 (11)
C12	0.59817 (14)	0.2425 (2)	0.1664 (3)	0.0462 (6)	
H12A	0.6022	0.3265	0.1678	0.055*	
O1'	0.48028 (12)	0.28051 (16)	0.3048 (3)	0.0661 (6)	
C2'	0.53057 (15)	0.1931 (2)	0.2458 (3)	0.0453 (6)	
C3'	0.50203 (17)	0.0833 (2)	0.2865 (3)	0.0532 (7)	
H3'A	0.5247	0.0088	0.2625	0.064*	
C4'	0.43150 (17)	0.1016 (3)	0.3722 (4)	0.0581 (8)	
H4'A	0.3989	0.0421	0.4144	0.070*	
C5'	0.42112 (18)	0.2205 (3)	0.3803 (4)	0.0677 (9)	
H5'A	0.3790	0.2580	0.4309	0.081*	

### Atomic displacement parameters (Å^2^)

**Table:**

	*U*^11^	*U*^22^	*U*^33^	*U*^12^	*U*^13^	*U*^23^
O1	0.0666 (14)	0.0566 (13)	0.132 (2)	0.0106 (11)	0.0528 (14)	−0.0037 (13)
S7	0.0568 (5)	0.0387 (4)	0.0861 (6)	−0.0057 (3)	0.0330 (4)	−0.0040 (3)
N1	0.0458 (12)	0.0368 (12)	0.0594 (13)	−0.0038 (9)	0.0206 (10)	−0.0035 (9)
C2	0.0426 (13)	0.0371 (14)	0.0465 (13)	0.0011 (11)	0.0084 (11)	−0.0033 (11)
N3	0.0448 (12)	0.0374 (12)	0.0562 (13)	0.0023 (9)	0.0121 (10)	−0.0027 (9)
C4	0.0450 (15)	0.0491 (16)	0.0630 (16)	0.0050 (12)	0.0176 (13)	−0.0004 (13)
C4A	0.0391 (13)	0.0477 (15)	0.0489 (14)	0.0007 (11)	0.0107 (11)	−0.0012 (11)
C5	0.0412 (14)	0.0499 (16)	0.0563 (15)	−0.0021 (12)	0.0144 (12)	0.0009 (12)
C5A	0.0466 (16)	0.068 (2)	0.092 (2)	−0.0008 (15)	0.0275 (15)	0.0024 (17)
C6	0.0466 (15)	0.0526 (16)	0.0615 (16)	−0.0084 (13)	0.0175 (13)	0.0020 (13)
C6A	0.068 (2)	0.0541 (18)	0.094 (2)	−0.0126 (15)	0.0291 (17)	0.0071 (16)
C7A	0.0421 (13)	0.0398 (14)	0.0543 (15)	−0.0018 (11)	0.0129 (11)	−0.0014 (11)
C8	0.0442 (14)	0.0357 (13)	0.0467 (14)	−0.0040 (11)	0.0061 (11)	−0.0004 (10)
C9	0.0596 (17)	0.0386 (15)	0.0645 (17)	−0.0044 (13)	0.0155 (14)	−0.0031 (12)
C10	0.065 (2)	0.0360 (17)	0.066 (3)	0.0019 (16)	0.010 (2)	−0.0066 (16)
C10A	0.048 (13)	0.032 (11)	0.10 (2)	0.004 (9)	0.008 (13)	0.013 (12)
C11	0.0681 (19)	0.0365 (14)	0.083 (2)	0.0089 (14)	0.0169 (16)	−0.0035 (14)
C12	0.0472 (14)	0.0337 (13)	0.0587 (15)	−0.0056 (11)	0.0115 (12)	−0.0006 (11)
O1'	0.0624 (12)	0.0378 (10)	0.1018 (15)	−0.0008 (9)	0.0407 (11)	0.0043 (10)
C2'	0.0444 (14)	0.0357 (13)	0.0567 (15)	−0.0030 (11)	0.0110 (12)	−0.0011 (11)
C3'	0.0576 (16)	0.0346 (14)	0.0692 (18)	−0.0072 (12)	0.0196 (14)	−0.0015 (12)
C4'	0.0539 (16)	0.0467 (16)	0.0760 (19)	−0.0121 (13)	0.0236 (14)	0.0027 (13)
C5'	0.0569 (17)	0.0501 (18)	0.100 (2)	−0.0057 (14)	0.0384 (17)	0.0024 (16)

### Geometric parameters (Å, °)

**Table:**

O1—C4	1.226 (3)	C9—H9A	0.9700
S7—C7A	1.732 (3)	C9—H9B	0.9700
S7—C6	1.748 (3)	C9—H9C	0.9700
N1—C2	1.321 (3)	C9—H9D	0.9700
N1—C7A	1.357 (3)	C10—C11	1.478 (5)
C2—N3	1.379 (3)	C10—H10A	0.9700
C2—C8	1.479 (3)	C10—H10B	0.9700
N3—C4	1.422 (3)	C10A—C11	1.41 (2)
N3—C11	1.482 (3)	C10A—H10C	0.9700
C4—C4A	1.435 (4)	C10A—H10D	0.9700
C4A—C7A	1.377 (3)	C11—H11A	0.9700
C4A—C5	1.449 (3)	C11—H11B	0.9700
C5—C6	1.352 (4)	C11—H11C	0.9700
C5—C5A	1.510 (4)	C11—H11D	0.9700
C5A—H5AA	0.9600	C12—C2'	1.437 (3)
C5A—H5AB	0.9600	C12—H12A	0.9300
C5A—H5AC	0.9600	O1'—C5'	1.363 (3)
C6—C6A	1.509 (4)	O1'—C2'	1.381 (3)
C6A—H6AA	0.9600	C2'—C3'	1.349 (3)
C6A—H6AB	0.9600	C3'—C4'	1.414 (4)
C6A—H6AC	0.9600	C3'—H3'A	0.9300
C8—C12	1.344 (3)	C4'—C5'	1.325 (4)
C8—C9	1.506 (3)	C4'—H4'A	0.9300
C9—C10A	1.51 (2)	C5'—H5'A	0.9300
C9—C10	1.531 (4)		
			
C7A—S7—C6	91.33 (12)	C8—C9—H9C	109.1
C2—N1—C7A	116.0 (2)	C10A—C9—H9D	109.1
N1—C2—N3	122.1 (2)	C8—C9—H9D	109.1
N1—C2—C8	117.8 (2)	H9C—C9—H9D	107.8
N3—C2—C8	120.1 (2)	C11—C10—C9	112.2 (3)
C2—N3—C4	123.4 (2)	C11—C10—H10A	109.2
C2—N3—C11	120.8 (2)	C9—C10—H10A	109.2
C4—N3—C11	115.7 (2)	C11—C10—H10B	109.2
O1—C4—N3	119.5 (3)	C9—C10—H10B	109.2
O1—C4—C4A	126.7 (2)	H10A—C10—H10B	107.9
N3—C4—C4A	113.8 (2)	C11—C10A—C9	117.6 (17)
C7A—C4A—C4	117.6 (2)	C11—C10A—H10C	107.9
C7A—C4A—C5	113.2 (2)	C9—C10A—H10C	107.9
C4—C4A—C5	129.2 (2)	C11—C10A—H10D	107.9
C6—C5—C4A	111.6 (2)	C9—C10A—H10D	107.9
C6—C5—C5A	124.3 (2)	H10C—C10A—H10D	107.2
C4A—C5—C5A	124.0 (2)	C10A—C11—N3	118.1 (9)
C5—C5A—H5AA	109.5	C10—C11—N3	111.7 (3)
C5—C5A—H5AB	109.5	C10A—C11—H11A	130.9
H5AA—C5A—H5AB	109.5	C10—C11—H11A	109.3
C5—C5A—H5AC	109.5	N3—C11—H11A	109.3
H5AA—C5A—H5AC	109.5	C10—C11—H11B	109.3
H5AB—C5A—H5AC	109.5	N3—C11—H11B	109.3
C5—C6—C6A	129.0 (2)	H11A—C11—H11B	107.9
C5—C6—S7	112.7 (2)	C10A—C11—H11C	107.8
C6A—C6—S7	118.3 (2)	N3—C11—H11C	107.8
C6—C6A—H6AA	109.5	H11B—C11—H11C	139.2
C6—C6A—H6AB	109.5	C10A—C11—H11D	107.8
H6AA—C6A—H6AB	109.5	C10—C11—H11D	138.9
C6—C6A—H6AC	109.5	N3—C11—H11D	107.8
H6AA—C6A—H6AC	109.5	H11C—C11—H11D	107.1
H6AB—C6A—H6AC	109.5	C8—C12—C2'	129.2 (2)
N1—C7A—C4A	127.0 (2)	C8—C12—H12A	115.4
N1—C7A—S7	121.74 (18)	C2'—C12—H12A	115.4
C4A—C7A—S7	111.21 (19)	C5'—O1'—C2'	106.6 (2)
C12—C8—C2	117.4 (2)	C3'—C2'—O1'	108.2 (2)
C12—C8—C9	123.0 (2)	C3'—C2'—C12	138.3 (2)
C2—C8—C9	119.6 (2)	O1'—C2'—C12	113.4 (2)
C10A—C9—C8	112.6 (8)	C2'—C3'—C4'	107.9 (2)
C8—C9—C10	111.1 (2)	C2'—C3'—H3'A	126.0
C10A—C9—H9A	135.2	C4'—C3'—H3'A	126.0
C8—C9—H9A	109.4	C5'—C4'—C3'	106.2 (2)
C10—C9—H9A	109.4	C5'—C4'—H4'A	126.9
C8—C9—H9B	109.4	C3'—C4'—H4'A	126.9
C10—C9—H9B	109.4	C4'—C5'—O1'	111.1 (2)
H9A—C9—H9B	108.0	C4'—C5'—H5'A	124.5
C10A—C9—H9C	109.1	O1'—C5'—H5'A	124.5

### Hydrogen-bond geometry (Å, °)

**Table:**

*D*—H···*A*	*D*—H	H···*A*	*D*···*A*	*D*—H···*A*
C3'—H3'A···O1'^i^	0.93	2.58	3.442 (3)	154
C4'—H4'A···N1^i^	0.93	2.66	3.568 (3)	166
C5A—H5AA···O1^ii^	0.96	2.55	3.486 (4)	166
C6A—H6AA···O1^ii^	0.96	2.62	3.571 (4)	171

Symmetry codes: (i) −*x*+1, *y*−1/2, −*z*+1/2; (ii) −*x*+2, *y*+1/2, −*z*−1/2.
